# Young People’s health-related learning through social media: What do teachers need to know?

**DOI:** 10.1016/j.tate.2021.103340

**Published:** 2021-06

**Authors:** Victoria A. Goodyear, Kathleen M. Armour

**Affiliations:** a School of Sport, Exercise and Rehabilitation Sciences, University of Birmingham, Birmingham, B15 2TT, UK; b Pro-Vice-Chancellor-Education, University of Birmingham, Birmingham, B15 2TT, UK

**Keywords:** Social media, Schools, Digital literacy, Wellbeing, Health-related learning, Professional development, School-based policies

## Abstract

International regulatory bodies have argued that young people should be better supported to engage safely, responsibly and effectively with social media. This paper considers ways in which the introduction of structured social media engagement in schools could bring educational benefits for young people, particularly in supporting them to deal with challenges relating to health and wellbeing. New evidence is provided on: (i) the value of social media as a health-related learning tool to bridge informal and formal learning contexts; (ii) how teachers should be supported to better understand and respond to young people’s learning needs; and (iii) the school-based policies, expectations and resources that will help teachers to offer relevant support.

## Highlights

• Social media is embedded in young people’s lives and is as an effective educational and health-related medium.
• Teachers and young people should co-construct shared understandings of social media and its impacts.
• Teachers need to be empathetic rather than antagonistic toward young people’s uses of social media.
• Teachers should positioning themselves as continuous learners willing to co-construct digital pedagogies with young people.
• The tendency for schools to ban mobile phones should be re-considered given educational and health-related benefits.

## 1 Introduction

The role of social media in the lives of contemporary young people is receiving significant attention in scientific, public, and policy arenas. Time-use data from the Millennium Cohort Study in the UK indicate that more than two thirds of young people (66%, avg age 14) use social media for at least 3 h per day and one fifth (21%) use social media for more than 5 h per day (Scott, Biello, & Woods, 2019). Similar findings have been reported in Europe and the USA (Eurostat, 2015; Pew Research Centre, 2018) and there is broad agreement that contemporary young people are avid consumers and producers of social media content (Third, Collin, Walsh, & Black, 2019), and that social media provides a vehicle for young people to engage with the world at large (Greenhow, Cho, Dennen, & Fishman, 2019). In turn, leading regulatory bodies across the world have argued that young people should be better supported to engage with social media safely, responsibly, and effectively. Examples include the OECD (2019), the UK Chief Medical Officers (Davies, Atherton, Calderwood, & McBride, 2019), the Children’s Commissioner in the UK (2018), the European Commission (2017), and The American Academy of Paediatrics (2015). Much of the debate has centred on social media sites, online harms and the need for better regulation and monitoring of content and use (c.f. Online Harms White Paper, HM Government, 2019; House of Commons, 2019). Less widely recognised is students’ *informal learning* through social media, the potential of social media platforms to offer *positive educational opportunities*, and how structured social media use in *schools* can relate to and support larger challenges in young people’s lives, such as those related to *health and wellbeing* (Carpenter, Tur, & Marín, 2016; Greenhow et al., 2019; Rutledge, Dennen, & Bagdy, 2019).

Social media can be broadly defined as a user-centric and interest-driven technology that is characterised by the distribution of user-generated information in self-selected social networks (Papacharissi, 2014). For young people, particularly adolescents, this medium is engaging and attractive because it aligns with key developmental behaviours, such as independence, risk taking and engagement and interest with peer groups (Blakemore, 2019). Indeed, social media provides vast opportunities for young people to pursue their interests and/or risky topics or groups, often with peers and mainly out of the direct view of adults (Ito et al., 2010). In turn, this level of privacy can be viewed both positively and negatively. For example, in the context of learning about health and wellbeing, one advantage of social media is that it offers relatively private spaces for young people to access information, in areas such as, physical activity, diet/nutrition and body image (Goodyear, Armour, & Wood, 2019). This can be particularly important when young people find health-related topics such as body image embarrassing and are unwilling to participate in offline communal physical activity contexts (e.g. physical education) (Goodyear & Armour, 2019; Freeman, Caldwell, & Scott, 2020; Wartella, Rideout, Montague, Beaudoin-Ryan, & Lauricella, 2016). At the same time, the potential for harm is also illustrated if young people are accessing false or inappropriate information. Either way, social media is a highly accessible resource for young people during adolescence when they tend to be curious and keen to learn about themselves and their bodies (Goodyear & Armour, 2019) . Finding ways to harness and manage this resource for positive educational benefit is, therefore, an important task.

It is well established that education and health are powerfully linked, and that schools support adolescents through developmental periods of social, emotional and physical changes (Bonell, Blakemore, Fletcher, & Patton, 2019). Yet young people’s online learning experiences tend to exist in parallel to formal learning experiences in schools (Halverson & Shapiro, 2013; Rutledge et al., 2019), and there are few examples of the ways in which teachers integrate their classroom practices with students’ informal learning about health through social media (Greenhow et al., 2019; Goodyear & Armour, 2019; Kirk, 2019). Social media tends to be cast as a distraction in schools (Selwyn & Aagaard, 2020), with the focus of technology integration mainly centred on accountability policies designed to measure and document learning, rather than support the learning needs and interests of young people (Halverson, Kallio, Hackett, & Halverson, 2016; Halverson & Shapiro, 2013). There is, therefore, a gap in research and policy that leaves many schools and teachers ill-equipped to support and enrich young people’s learning through and from their extensive social media engagement.

To understand how teachers can enrich and support young people’s informal learning through social media, this paper examines health-related social media policies and practices in UK schools, and considers the perspectives from young people, teachers and key stakeholders in education and health (researchers, professionals and practitioners). In this paper we adopt a broad conceptualisation of health as physical, social and mental wellbeing (World Health Organisation, 2006). Health and wellbeing are therefore intertwined and grounded in an individual’s perspective on his or her physical, psychological and social state of being (World Health Organisation, 2006). Accordingly, health-related social media refers to forms of social media content (e.g. information, memes, videos, stories) and/or uses of social media (e.g. to consume and/or produce content) that can inform physical, social and emotional wellbeing. To provide clarity for participants (young people, teachers and key stakeholders), data collection was framed initially by a focus on health-related social media in the areas of physical activity, diet/nutrition and body image. Overall, the aim of this paper is to find ways to enhance young people’s formal and informal learning and, as a result, support them to improve their health and wellbeing.

## 2 Literature review

It is evident that social media engagement is a daily and informal (potential) learning activity for many young people. The earlier work of Ito et al. (2010) illustrated that young people learn with social media via observation and communication in self-selected friendship-driven and interest-driven networks. More recently, it has been argued that the interactive functionalities of social media, that enable interaction, collaboration, information access, and resource sharing, facilitate an array of learning activities, such as: collaborative knowledge construction, the hybridization of expertise, relational development, peer support, and social and civic learning (Carpenter & Krukta, 2014; Greenhow & Askari, 2017; Greenhow & Lewin, 2016; Krutka & Carpenter, 2016). Evidence suggests that young people are using these networks and interactive functionalities for active and self-directed learning that is largely separate from schooling, and is often focussed on career development, building knowledge and interests, and learning new skills (Greenhow & Askari, 2017; Rutledge et al., 2019). There is also clear evidence that young people access information on social media to inform and improve their health-related knowledge and behaviours, and in areas related to physical activity, diet/nutrition and body image (Goodyear, Armour, & Wood, 2019; Wartella et al., 2016). Gender differences in how young people learn with social media have also been observed (Booker et al., 2018; Scolari, 2018). In relation to physical activity and body image , it has been argued that girls tend to learn through social media stories, and micro-pedagogical practices of judgement and comparison (Goodyear, Andersson, Quennerstedt, & Varea, 2021) and boys tend to learn through interactions associated with hobbies and interests, and through micro-pedagogical practices of irony and humour (Goodyear & Quennerstedt, 2020) . This evidence of young people’s informal learning using social media provides interesting insights into the ways in which learning could be organised and structured in formal learning contexts (Greenhow & Askari, 2017; Greenhow et al., 2019).

Given the vast opportunities for learning afforded by social media engagement, numerous interdisciplinary researchers and stakeholders in education and health have argued for digital engagement to be conceptualised as a fundamental right for young people (Livingstone & Third, 2017; Third et al., 2019). From this rights-based perspective, it is argued that a focus on media literacy in schools will assist and empower young people to engage with society effectively through media (Jenkins, Purushotma, Weigel, Clinton, & Robison, 2009; Livingstone et al., 2017; Third et al., 2019). As a result, young people can develop the skills and knowledge they need to engage effectively with social media, and the skills to determine what information is useful, misleading, trusted and/or stems from specific political or commercial interests (Livingstone & Third, 2017; Third et al., 2019). Similar arguments have been made in a health context, with a focus on digital health literacy positioned as a productive means by which to assist young people to realise the potential benefits of social media for health and wellbeing (Dudley, Bergen, McMaugh, & Mackenzie, 2019; Rich, 2019). Yet there is an emerging trend across international contexts for mobile phones and social media to be prohibited in schools and classrooms (Rutledge et al., 2019; Selwyn & Aagaard, 2020). These school policies are based on popular assumptions that restricting phones and social media use will improve educational attainment and wellbeing, and reduce the prevalence of addictive behaviours and cyberbullying (Berger, 2017; Selwyn & Aagaard, 2020), even though there is little evidence to support these assumptions (Beland & Murphy, 2015; Kvardová, Valkovičová, & Šmahel, 2019), and the evidence suggests the risks for young people online are relatively low (Livingstone & Third, 2017). Overall, school phone ban policies are a useful illustrative example of Halverson and Shapiro’s (2013) argument that legal, moral and practical issues persistently thwart teachers’ attempts to integrate aspects of students’ social lives into formal learning experiences in meaningful ways. Furthermore, school policies - like phone bans - tend to be in conflict with a digital rights perspective.

Although the school environment tends to be guided by restrictions on social media use, investigation into classroom based integration of social media is one of the most common topics for research in this field (Greenhow & Askari, 2017). Studies focus on the impact of social media on educational outcomes and the process of implementation (Greenhow & Askari, 2017). Examples include: the use of Ning to develop students’ higher order thinking (Callaghan and Bower (2012), and the use of Facebook in Biology to develop students’ literacy skills (Veira, Leacock, & Warrican, 2014). Yet, there are relatively few examples of social media being used strategically in the classroom (Greenhow et al., 2019) and many teachers report needing further training on how to utilize social media as a learning tool (Fewkes & McCabe, 2012; Kale & Goh, 2014). It is certainly apparent that there is a gap in the extent to which students use social media and teachers’ understanding of the medium and its affordances (Rutledge et al., 2019), and this is problematic in the process of classroom implementation (Greenhow & Askari, 2017; Kale & Goh, 2014). Furthermore, while classroom implementation studies provide valuable evidence on social media use in formal learning contexts, they are often constrained to a focus on pre-defined educational outcomes (Greenhow et al., 2019). As a result, they fail to tap into the vast intrinsic motivation evident in the ways in which students explore online tools and their affordances for learning (Rutledge et al., 2019). Greenhow et al. (2019) argue that this approach to research fails to account for the disruptive and pervasive nature of social media in young people’s lives, and limits opportunities to learn about how this could (and does) enrich learning within and beyond school.

There is remarkably little research on the links between social media use in and as part of school-based learning, and its impacts on young people’s informal learning outside of school (Carpenter et al., 2016; Greenhow et al., 2019; Rutledge et al., 2019). This is surprising given the evidence on young people’s extensive access to and use of social media, and the cross-cutting potential of this medium to be used in a fluid and dynamic manner across learning contexts and in young people’s lives. Furthermore, there is a broad evidence-base in the field of public health on the positive impact of health-based social media interventions on health-related knowledge, attitudes and behaviours (Hamm et al., 2014; Müller, Alley, Schoeppe, & Vandelanotte, 2016). For example, systematic reviews demonstrate the potential of social media interventions to improve children and young people’s physical activity knowledge, attitudes and behaviours and enhance the quality of their diet, including some interventions that have been applied to school settings (Chau, Burgermaster, & Mamykina, 2018; Hsu, Rouf, Diet, & Allman-Fairnelli, 2018). However, it appears that formal education contexts have attempted to ignore or dismiss the potential of social media as a health-related learning tool, perhaps because it is perceived to be ‘in the hands’ of young people themselves. Yet, for many young people, social media is accessible, pervasive and alluring, so it is timely to think differently about its educational potential and to find new ways to support the use and development of effective school policies and practices (Greenhow et al., 2019; Halverson et al., 2016).

## 3 Theoretical foundations

In order to support the development of effective school policies and practices that can exploit the educational potential of social media, research must be grounded in the complexities of the school environment, and the challenges of integrating informal and formal learning contexts (Greenhow et al., 2019). Socio-cultural perspectives on learning explicitly account for fluid connections and disruptions between different contexts for learning, so they provide an appropriate theoretical framing for this research.

From the broad array of socio-cultural theories, Public Pedagogies (Burdick & Sandlin, 2013) and Participatory Cultures (Jenkins et al., 2009) were selected for this research because they foreground learning as an *experience* that is influenced by culture and not confined to singular and/or formal educational spaces (Burdick & Sandlin, 2013; Jenkins et al., 2009), and so have the explanatory power required. Importantly, both can accommodate the understanding that health and learning with social media cannot be abstracted from other aspects of young people’s lives (Giroux, 2004; Rich & Miah, 2014) because learning (including health-related learning, and from young people’s perspectives) is happening simultaneously across media, home, school, and community contexts (Goodyear & Armour, 2019; Greenhow, Robelia, & Hughes, 2009). To be effective in supporting young people’s learning with social media, schools would need to take into account these vast learning ecologies (Greenhow et al., 2009), and develop context-dependent forms of health and learning that take into account *experiences* and their relationships to culture (Giroux, 2004).

Public pedagogies focus on how various sites, spaces, products and places in our daily lives operate pedagogically (Burdick & Sandlin, 2013). Often grounded in the work of Giroux (2004), pedagogy is interpreted as a political and moral practice. As a political practice, pedagogy illuminates the relationship between power, knowledge and ideology. As a moral practice, pedagogy recognizes that what the media teaches cannot be abstracted from what it means to invest in public life; for example, to locate oneself in public discourse. Accordingly, public pedagogies is described as the regulatory and emancipatory relationship between culture, power and politics that occurs in a democratically configured social space. Similarly, participatory cultures take an ecological perspective on learning, and rather than focus on technology in isolation, a participatory culture emphasises the interrelationship between different communication technologies, cultural communities, and the activities they support (Jenkins et al., 2009). Often grounded in the work of Jenkins, a participatory culture can be defined as a ‘culture with relatively low barriers to artistic expression and civic engagement, strong support for creating and sharing one’s creations, and some type of informal mentorship whereby what is known by the most experienced is passed along to novices’ (Jenkins et al., 2009, p.xi). Through the lens of both public pedagogies and participatory cultures, it can be argued that far from being ignored or vilified in the classroom, social media should be recognised as a powerful part of the hidden curriculum and one that is fully integrated in students’ learning (Rosen, Carrier, & Cheever, 2013).

The gap between learning in schools and learning through social media is evident in the differing learning experiences that these contexts engender (Halverson et al., 2016). Technologies such as social media are organised to serve the needs of users, providing them with autonomy and control over accessing information the user considers to be relevant (Halverson et al., 2016; Jenkins et al., 2009). By contrast, schools are primarily organised around pre-determined and standardized learning experiences with a focus on the mastery of set content and skills (Halverson et al., 2016; Jenkins et al., 2009). As cultures are grounded in established ideas and practices, the school culture tends to plays an ‘inherently conservative role of situating new practices in terms of existing norms, values and routines’ (Halverson & Shapiro, 2013, p. 16). Hence, to understand how schools could accommodate young people’s learning with social media, it is necessary to understand how learning with social media can be integrated effectively within the culture of schooling (Halverson & Shapiro, 2013; Rutledge et al., 2019).

This integration or bridging challenge is not new. The work of John Dewey (1916), for example, has been widely cited on this topic and it has contemporary relevance in seeking to identify the types of school policies and practices that would be effective in bridging the gap between school-based learning and that taking place informally through social media. In particular, Dewey argued that education is grounded in experience and it is the ‘reconstruction or reorganization of experience which adds to the meaning of experience, and which increases the ability to direct the course of subsequent experience’ (Dewey, 1916, p. 76). From a Deweyan perspective, schools are one dimension of public pedagogy and one site where the reorganization of experience occurs (Schubert, 2010). In particular, Dewey (1938) proposed that educational research should interrogate the distinction between education and miseducation. Miseducation is described as what occurs when experiences evoke discord rather than harmony, monotony rather than variety, and are constraining instead of expansive (Dewey, 1938). Furthermore, Dewey (1938) argued that in order to be educative, schools need to recognise implicit and explicit curriculum learning experiences across disciplines of knowledge, acknowledging that much of what students learn in schools is in addition to what educators intend, and that extensive learning occurs beyond schools. Hence, Dewey (1938) advocated for schools to import learning from the broader social milieu of life to shape educational experiences.

Overall, perspectives from the work on public pedagogies, participatory cultures and Dewey suggest that, to be effective, school policies and practices should find ways to acknowledge and extend the learning that is already taking place through young people’s extensive engagement with social media. Accordingly, to better understand how teachers can harness, enrich and support this informal learning, the purpose of this paper is to identify the connections and points of difference between young people’s health-related learning needs and interests and school-based social media policies and practices. The following research questions guided this research.

In relation to school policies and practices associated with social media with a particular focus on health-related learning:

1. Are the interests of learners considered in initiating health-related learning experiences?
2. Are health-related learning experiences reconstructed and relative to young people’s on-going experiences with social media?
3. What health-related learning experiences in schools are envisaged as expansive rather than constraining?

## 4 Methods

### 4.1 Research design

The participants and data reported in this paper are part of a wider participatory action research project examining young people’s uses of health-related social media. Data collection took place over 2 overarching phases. Phase 1 aimed to determine how young people develop health-related knowledge, skills and behaviours through their uses of social media (see Goodyear, Armour, & Wood, 2019). Phase 2 aimed to determine how schools and teachers can harness, enrich and support young people’s informal learning by identifying points of difference between young people’s health-related needs and interests and school-based social media policies and practices. Hence, the data reported in this paper is related to phase 2, and for details of the wider project please see Goodyear & Armour, (2019).

### 4.2 Ethics

A culturally responsive relational and reflexive approach to ethics was adopted , meaning that care for participants involved creating and using data collection methods that ensured participant safety, privacy, and dignity, and that promoted participant autonomy (see Goodyear & Armour, 2019for further detail). Ethical approval was provided by the University STEM ethics committee and informed consent or assent was sought.

### 4.3 Participants and setting

There were 170 participants in total and these included young people (n = 135, age 13–18), teachers (n = 9), community workers (n = 6), and researchers (n = 20) specializing in school-based policies and health interventions. These diverse and multiple participants were selected to provide a holistic understanding of the connections and differences between young people’s learning through social media and existing school policies and practices, and to incorporate knowledge from diverse school contexts on the types of policies and/or practices that could be considered by schools/teachers. Data were collected in the UK between 2016 and 2017. Young people and teachers were recruited from 10 schools that were engaged with the wider project. The schools were located in the West Midlands and the South of England. Of the 10 schools, 2 were private, 3 were government-run and 5 were academies, 2 of which were faith schools. The schools were located in diverse socio-economic areas and included students from a range of different ethnic backgrounds, with just under a third of students across these schools speaking English as a second language. Community workers represented professionals from organizations (e.g. local council/authorities, charities, trusts) that offer educative services to schools and work with young people in community settings to support health and wellbeing. Community workers were recruited either from the schools engaged with the research and/or through the University of Birmingham’s public engagement and public affairs teams. Researchers were recruited from the research teams’ professional contacts, and were selected due to specializing in school-based policies and/or health interventions, and being capable of offering evidence-based perspectives from diverse school contexts.

### 4.4 Data collection

A multi-method approach was used to engage with multiple and varying groups of the participants at different stages of the research.

*Young people*: Data were collected from focus group (FG) interviews and a workshop. The aim of the FGs was to identify the types of school policies and practices that young people currently experienced and perceived to be relevant and supportive of their health-related uses of social media (RQ1, RQ2). Participants for the FG were recruited as part of the wider study and from the 10 schools. 19 FGs (n = 84; age 13–15; m = 35; f = 49) were completed using elicitation techniques (e.g. images of health-related content of social media -as prompts) and semi-structured interview questions that were common across all of the FGs. The FGs were voice recorded and later transcribed. The workshop had the aim of identifying what types of school policies and practices could enrich young people’s learning with social media (RQ3). The workshop took place with 51 young people (m = 32; f = 19) from 5 of the 10 schools in the wider study (who volunteered to participate). During the workshop young people worked in small groups (n = 5) to create guidelines and resources for their schools to help their teachers’ better support their health-related uses of social media. Data were collected from transcribed text from the resources young people created and these included radio and TV interviews, podcasts, movies, and newspaper articles.

*Teachers, Community Workers and Researchers:* Data were collected during a 1-day seminar. The aim of the seminar was to understand, from the perspectives of teachers, community workers and researchers, existing school policies and practices on health-related social media, the context and reasons for using these (RQ1, RQ2), and whether different policies and practices might be needed and/or required to enrich and support young people’s learning through social media (RQ3). In total data were collected from 35 participants, and these included teachers from the participating schools (n = 9; m = 2; f = 7), community workers associated with the participating schools (n = 6; m = 4; f = 2), and researchers specializing in school-based policies and interventions (n = 20; m = 11; f = 9, UK = 12, International = 8). The participants of the seminar worked in small groups (n = 7) to complete several discussion-based tasks and data were collected from video recordings of these discussions.

### 4.5 Data analysis

A deductive theoretical thematic analytical approach (Braun & Clarke, 2006) was applied and took place in three main steps using the coding process outlined in Table 1.

**Table 1:** An illustration of the process of coding.

Codes	Categories	Themes
Democratic	Autonomy vs Control	Interests of Learners (RQ1)
Independent		
Accessible		
Priorities		
Risk	Teachers’ knowledge of social media	Continuous Reconstruction of Experience (RQ2)
Outdated		
Relevance/Irrelevance		
Outsourcing		
Digital Literacy	Collaborative Expertise	Expansive School Practices and Policies (RQ3)
Experiences		
Constructivist/Inquiry		
Experts		

Step one of data analysis involved organization of and familiarization with the data (Braun & Clarke, 2006). The data sets were read through by the researchers to identify specific aspects of the data that related to the three research questions. This process ensured that the research questions remained a central focus while also remaining open and reasonable to emerging understandings.

Step two involved the construction of themes. To generate initial codes (Braun & Clarke, 2006), the data sets were approached deductively using the research questions (see Table 1). The codes were then collated to identify potential patterns. This phase of analysis was also informed by the selected socio-cultural theoretical framework, and in particular Dewey (1938), whereby the collated codes were interrogated in relation to evidence of: ‘*discord vs harmony*’, ‘*monotony vs variety*’ and ‘*constraining vs expansive’.*

In the third step, a deliberative strategy, inspired by Tracy’s (2010) end goals for excellent qualitative research, was used. Each of the three researchers involved in the wider project had formulated categories from the initial coding process in response to each research question (Table 1). These categories became the basis for deliberation, and were discussed until consensus was reached. The codes, categories and their representations under the research questions are presented in Table 1.

### 4.6 Quality measures

A relativist approach to validity aimed to extend the robustness of traditional measures of quality (Smith & McGannon, 2018). As such, a list of characterising traits were selected, in relation to their relevance to the study and its question, design, and data collection techniques. The following traits were included: the worthiness of the topic; the significant contribution of the work; width – meaning the comprehensiveness of evidence from a wide sample of participants from diverse contexts, as well as multiple and in-depth data collection methods; credibility, through the iterative phased design and analytical process constructed between the researchers regarding the fairness, appropriateness and believability of the interpretations offered; and coherence, reflecting the ways in which the study’s purpose, theory, methods and results are aligned.

## 5 Results

The data generated from young people highlight a series of deep contradictions that must be navigated if teachers are to be successful in supporting young people and optimising the health-related educative potential of their extensive social media engagement. The overriding message is that teachers will be challenged to operate at the very boundaries of the intrinsically dynamic virtual spaces that characterise social media, while also dealing with young people in the traditional physical spaces of the school/classroom. On the other hand, despite gaps in understanding and experience, the data highlight clear areas in which both teachers and young people can collaborate to offer specific areas of expertise. For example, the data show that young people have specific forms of expertise in social media and that teachers have specific forms of expertise in young people’s health and development, and in relation to their physical, social and mental wellbeing. The data suggest that in order to support teachers, schools need to provide opportunities for teachers and young people to collaborate in order to develop shared understandings and skills about social media, and to co-construct relevant forms of support to optimise the positive learning potential of social media, and to mitigate any risks.

### 5.1 Interests of learners – autonomy vs control

There was agreement across the sample that social media use was a valuable health-related learning resource for young people, but that informal learning with social media was not a priority focus for school policies and practices. Firstly, the data showed broad support for social media in the lives of young people because it was a relevant and accessible source of health information that could cater for young people’s individual needs and shared interests.

We know that one of the most important factors for health and wellbeing is democracy, living in a democratic society where people can speak up. Okay so give them that tool. That is a tool for health … So, digital media can be a fantastic place for democratic communication (Seminar, Researcher, Male).

Say I wanted to be more healthy. Not healthy, but like lose weight or something. I type in to Google or YouTube or something, and then loads of YouTubers post like fitness videos and their routines and stuff and like five minute ones or something, or like 20 minutes and you just like do that. But I mean, I don’t think that’s too bad, because they’re not saying you have to, they’re just providing it there in case you want to, because you’ve had to type in and find it (FG Interview, School 8, Male).

In light of the perceived value of social media as a health-related learning tool outside of school, the young people and their teachers reported that social media should be used as a pedagogical tool in schools. Both teachers and young people agreed that social media was as an engaging and accessible medium that could support independent learning. The value for young people was grounded in the speed, accessibility and accuracy of information, particularly in comparison to ‘traditional’ educative resources that were used predominantly in schools (e.g. textbooks).

I think teachers are always taught that learning is maximised when students engage with what’s going on, so if they can relate the learning to them, they’ll learn more, so if you can have Twitter, and Snapchat and things in the lessons, they are more likely to enjoy it, and learn more from it (Seminar, Teacher, Male).

Students from a local school have recommended that throughout the school day any technological device – including social media – should be allowed. The reason for this is in a lesson we could be able to research any information to help us quick and easily, as it’s an everyday tactic. For example, in History you can find out information that happened back in a century and now. Another point is that text books in many lessons can be out of date so it’s false information. In addition to this, the students find writing and copying out of a text book boring so allowing phones and social media creates a positive atmosphere and they can be happy. Textbooks are a lot more fidgety than a technology device as the pages are annoying and trashy (half the textbooks are missing pages) so using social media can help students learn and make them more independent (Young People Workshop, Newspaper Article).

Despite the perceived value of connecting informal and formal learning experiences, existing school policies and practices were not conducive to harnessing social media. Instead, the challenges of integrating young people’s formal and informal learning experiences with social media were noted, where the easiest option was to ban social media and prioritise other learning agendas for which schools were held externally accountable. Teachers and community workers reported that infrastructures outside of schools (such as government policy and guidance) could help schools to address social media use and cater for contemporary young people’s learning needs and interests.

Schools at the moment are scared, so they’re kind of at a crossroads. We’ve worked together and get kids to use their phones and iPads and things in lessons, and it works really well, but since then our school’s completely banned phones. They’re not allowed to use their mobile at all. Because they’re scared. They don’t know what’s going on their phones while in school so it’s easier for them to put a blanket ban on it. And schools are like, do we allow it, do we embrace it, do we engage students with it, or do we just completely ignore it? (Seminar, Teacher, Male).

Schools’ priority would be assessment data, it would be safeguarding and this [social media] would keep getting sort of pushed down there because it would really matter in the long run, well it would matter in the long term for the students, but it is not what Oftsed^1^ would come in and see. They want safeguarding, they want data, they want attendance. (Seminar, Teacher, Female).

Researcher (male): Education of all parties is needed. Education of students, teachers and parents. Some kind of guidelines, it’s this idea of soft power

Teacher (male): I think everyone is still playing catch-up with it [social media]. It’s just kind of exploded. Schools are still playing catch-up.

Researcher (male): Very much so.

Teacher (male): Every time we’ve got to teach something, something changes and there’s different advice, so it’s about the government buying into social media and helping schools, this is what you need if you’re teaching them, and helping us to dedicate time for the 21st century.

Researcher (male): It changes very quickly and schools aren’t very agile. It takes a while for them to get onto things. By the time they’ve changed, everything changes again. So the idea of being kind of on the ball, on the finger, being proactive rather than reactive or something (Seminar, Group 6).

Although it was apparent that the democratic nature of social media is valued, this level of uncertainty tends to result in schools defaulting to banning young people’s engagement with social media during school hours.

### 5.2 Continuous reconstruction of experiences – teachers’ knowledge of social media

The findings revealed that although some schools resisted acknowledging young people’s extensive engagement with and informal learning from social media, existing school policies and practices did tend to address young people’s risk-related uses of social media. The content of these policies and practices - for example safeguarding and privacy – was, however, perceived by young people as being outdated and irrelevant.

When we get assemblies and stuff on social media it was always like the same thing. It’s like, put your privacy settings up. But nobody really cares anymore, because all they say is stuff about posting pictures and videos and privacy. They don’t talk about anything else that matters these days. Peer pressure, proper peer pressure is a bigger problem than cyberbullying (FG Interview, School 1, Female).

We believe that every bad thing that occurs in school should not be blamed on social media. This is because adults don’t understand how to use the applications (Young People Workshop, Newspaper Article, School 1).

The challenge of implementing relevant school policies and practices was attributed to a lack of teacher knowledge about social media and its contents. It is noteworthy that teachers acknowledged their tendency to focus on risks and also their lack of understanding about social media as a medium. Teachers were able to identify these issues as key factors hindering their capacity to understand and engage with young people about the ‘real’ risks they experience.

I don’t think that a lot of teachers here can really help. Like I have ‘Miss Smith’ and she’s just, I don’t suppose they all know a lot about social media (FG Interview, School 2, Male).

Researcher (male): Now I don’t profess to know much about social media, but how can we help young people who have fallen into a trap where they’re posting selfies, get 80 likes, and then all they can really think about is what will be there next photo …

Female (teacher): The answer lies in more knowledge of adults actually to detect those kind of problems in the first place, and then be able to talk about and invite the students into discussions, and that’s what is lacking (Seminar, Group 2).

I think we shouldn’t put our judgement on young people. We shouldn’t use our own fears and anxieties as reasons to suppress or stop young people from using these things. But nor should we be naïve enough just to let go with no help. So as we teach them … We’re assuming that this is a simple process and I think one of these things is that whole analogy about, we spent an awful lot of time, all of us as young people learning to walk. But we don’t remember that process of learning to walk. But we were there to support them (Seminar, Teacher, Female).

Despite clear gaps in teachers’ knowledge about social media and perceived inadequacies in the support they feel they can offer, the young people reported that being able to access relevant advice and guidance from teachers at times of vulnerability was essential for their wellbeing. For example, “say if someone had troubles, making sure that they know that they are able to go to someone to talk about it, and make sure that person has an idea and knows how to help” (FG Interview School 8, Male). The data point to an expectation by young people that teachers should develop their knowledge about social media and its risks and opportunities, in order to be more empathetic and less judgmental. For example: “if they [teachers] are made more aware of the real risks, then they can check in to see if you are struggling with those risks” (FG Interview, School 3, Male); “they need to know what they are talking about” (FG Interview, School 6, Female); “they need to be better informed about the problems of our generation” (FG Interview, School 8, Male).

The adults in the sample agreed that teachers needed to move from risk-related practices and aim to focus on experiences relevant to young people. There was evidence that some teachers’ current practices were relatable to young people’s experiences of social media, and these teachers reported that bridging offline health-related learning and health-content accessed by young people through social media was an effective means to simultaneously address risk while enriching engagement and learning. Other teachers reported that it would be more appropriate and effective for social media use to be addressed by external community workers who could deliver ‘guest’ or ‘expert’ talks and lessons.

I think it is getting away from this ‘it’s bad’, it’s something they do and it is something, like any other behaviour that carries its risks. It may be slightly different but it’s no different (Seminar, Researcher, Male).

“This Girl Can” campaign [national UK social media campaign] was fantastic for raising the profile of females in sport, but to some extent it wasn’t tangible for the pupils. So, for us, what we did is we did our own “This Girl Can” video and we used the role models in different year groups and we didn’t necessarily use the most able sportspeople in school because to some extent that was the top 2%, which are the intangible for the majority of the population in the school (Seminar, Teacher, Female).

I think they need real life experiences … we had a lady come in and speak about video gaming … and it really struck a chord with all of us … because it is very well us saying “don’t do this, don’t do that”. And it coming from a teacher. But actually it coming from her it was really powerful (Seminar, Teacher, Female).

Young people, however, reported that outsourcing social media support needed to be carefully considered to ensure that individuals with relevant experience and advice were selected to deliver talks; for example:

If we have a social media talk in school, from a police person who takes it to the extreme, their view of what we do is in stark contrast to what we actually do (FG Interview, School 6, Female).

Male 1: We normally get talks about social media and how we use it, not normally health-related, just like how to approach situations and stuff like that

Male 2: When they give talks on social media, the teachers don’t always fully understand it, so sometimes we don’t take it seriously

Male 1: It’s got to be relevant

Male 3: Someone who actually uses it

Male 2: A young person. That may be a bit discriminative, but if I saw someone, subliminally, I would listen to them more if they were younger, like 25

Male 1: Once there was a guy who suffered from a food disorder and from over exercising and he was actually from our school. He came to talk to us about food disorders. I thought that was quite good because it was actually someone we knew

Male 2: Yeah it needs to be someone believable (FG Interview, School 8).

Overall, the data from all participants pointed to a problematic gap in teachers’ knowledge and understanding of social media and its contents. Similar to the first theme, if social media is to be optimized in formal learning contexts, there needs to be a much better shared understanding between teachers and young people about social media. There is strong evidence to suggest that teachers need to be more knowledgeable so they can be empathetic about the multiple and extensive ways in which young people use social media and, when this is not possible, individuals with relevant experience of social media should be considered.

### 5.3 Expansive school practices and policies - collaborative expertise

The previous two themes identified and explained the types of school-based policies and practices that constrain how teachers’ support young people’s learning through social media. This theme explains the types of practices that are envisaged as expansive, and highlights the ways in which young people and teachers have very different yet relevant and specific forms of expertise that could be combined to ensure teachers can better support young people to be effective learners through their engagement with social media. Firstly, it was also notable in the data set that young people were positioned as the ‘teachers’ in digital literacy:

Female (Community Worker): I think the main issue is the digital literacy of the adults.

Female (Teacher): Yeah, because we don’t understand; we’re not involved in this.

Female (Community Worker): It’s totally different worlds, and actually normally when we talk about digital, we’re mostly talking about children, but in this case they are the experts, and the teachers don’t know, the parents don’t know. So I really think that’s really something to work on, because they can … What I know from media facts, is that it’s really important that the parents and teachers participate in what’s going on and talk about it. (Seminar, Group 5)

I think if people see other people their age telling them that this is … I think you’re most likely to listen to advice from your friends. So, if you’re listening to people that are your age, and are doing similar things, then you’re more likely to listen (FG Interview, School 3, Female).

In suggestions that come full circle back to the classroom, the participants suggested that teachers should proactively use social media as a pedagogical tool in their classrooms. In particular, constructivist pedagogies, such as critical inquiry tasks, that build on young people’s knowledge and experiences were reported as potentially effective strategies for bridging the gaps in teachers’ and young people’s understandings of social media, and the potential for positive learning experiences.

It’s a pedagogy of constructivist learning as it is in any other of getting young people to engage with the media actively and critically, getting them to go through their Instagram account and identify the first 10 adverts, and getting them to say, “So who do you think put it there? Why do you think they put it there? You discuss. What do you think? Are they trying to … What do they want you to do? How do you feel about that?” (Seminar, Researcher, Male)

We should focus on children, and making them a sort of taskforce so that they can teach the older people what they’re actually learning. So there’s a teaching approach from the bottom up, rather than from the top down. Most kids are more critical than the adults are. Because they actually know it’s fake, most of it. And that for me is a resource … What we need to do is give them more tools to unpack these. Because 20 years ago, we didn’t know if a picture was Photoshoppped or not. Now the kids know every picture is Photoshopped. And they know that. They use it anyway but they know its fake. And they know that there is fake information and not fake information. They know that research says different things about climate, change, diet or whatever. It’s more like, how can we give them as many tools as possible? And education is about giving kids tools that they will use at some point (Seminar, Researcher, Male)

In terms of what teachers can offer, the data from the young people highlighted teachers’ expertise in *content*. The young people reported that teachers could use their experience and knowledge to help them to identify fake or untrue posts, understand what information to use or disregard, and/or the types of posts could be beneficial or harmful. In particular, physical education teachers were identified as a key resource in accessing and using health-related social media. Young people clearly need help in determining the personal relevance – for themselves and in relation to their particular body concerns - of the plethora of available health-related social media content, and in identifying positive choices that will support their health and wellbeing.

Female 1: PE teachers could help you to know which ones [videos] to watch and what ones to do, because we don’t

Female 2: Know which ones are like age appropriate (FG Interview, School 6).

I would want support to come from a PE teacher or someone who’s involved in the world of fitness, health and sport … to make sure that person knows how to get fit and also knows how to help them deal with it if they’re feeling inadequate or feeling like they’re not fit enough … whereas if another teacher said it, you would think “He’s got a history degree, what does he know” (FG Interview, School 8, Male).

I know you can’t really tell people to stop posting and things like that, but they should have maybe a thing at school to … like an actual before and after difference because most of them are fake, aren’t they? (FG Interview, School 2, Female).

In addition to teachers’ expertise in social media content, the young people positioned teachers as a central resource in helping them understand the moral and ethical consequences of their behaviours on social media. Social/emotional learning about the potential impacts of young people’s behaviours on social media was reported to be an essential component of school education and an area in which teachers have much to offer:

I think the teacher should look at how posting one picture can make someone feel insecure about themselves. Because I will post a picture but I would never know how ‘Gemma’ really feels about it. You need to learn what the person who is looking at that image will feel because I might personally be completely different to somebody else, but the person that posted that picture doesn’t know how they feel (FG Interview, School 9, Female)

Female (teacher): We need to help young people to extend their ethical behavior to the digital context in which they are using, and we should help them to proactively shape their digital futures … it should be cooperative construction, that we would see as a process of co-construction between teachers and students

Male (researcher): As a democratic process

Male (community worker): Co-participation

Male (researcher): I think we need to advocate for that. To open the doors. So we’re not speaking to literacy and language. We’re not using different languages that we learn from each other, so we’re building a shared understanding (Seminar, Group 5)

Data in this theme suggest that young people are a central resource for teachers in developing understandings about how to support young people to learn through social media. The data pointed to young people’s specific forms of expertise in social media that could be harnessed to support: (a) young people’s uses of social media through peer-based learning/support strategies; and (b) teacher knowledge and understanding of social media as a context for learning. Equally, young people suggested that they looked to teachers for support. Young people felt that teachers could help them to determine the forms of content to access and act on, and how to make judgements about the actions of others on social media. Schools must therefore be in a position to support teachers and young people to collaborate around issues and opportunities related to social media.

## 6 Discussion

This study provides evidence on school policies and practices that enrich, support and/or hinder young people’s informal learning through social media. New evidence is provided on: (i) the value of social media as a health-related learning tool to bridge informal and formal learning contexts; (ii) how teachers can be supported to learn continuously to understand and respond to young people’s dynamic learning needs; and (iii) the school-based policies, expectations, and resources that will help teachers to offer relevant support, including at times of vulnerability. Overall, the findings illustrate that school-based policies and practices have the potential to enrich and support young people’s informal learning with social media, but such policies and practices need to be relatable to young people’s informal engagements and experiences with social media. The important point to make is that the central starting point for teachers in determining how to support young people should be a focus not on the technology, but on young people and their learning needs. More specifically, by focusing on what social media enables in young people’s learning, teachers can generate the knowledge they require on how and why young people do what they do on social media and what support they need to optimise educational benefits and mitigate risks. The key challenge, therefore, is to help schools and teachers better design social media policies and practices in ways that acknowledge the potential of social media to enrich learning across formal/informal contexts, and move beyond the positioning of social media as ubiquitously harmful and/or risky.

Teachers tend to be critiqued for their lack of knowledge and understanding about young people and social media (Galvin & Greenhow, 2020; Saunders et al., 2017). On the contrary, evidence from this study illustrates that teachers have valuable pedagogical expertise to add to this dynamic digital learning space, with the potential to engage with young people in developmentally and critically informed ways that could bridge intergenerational gaps. Although it is evident that many teachers lack specific skills in social media (Galvin & Greenhow, 2020), they do have important expertise in young people’s development, and this could be better utilised to decide when and how to support young people, and also how, when and with whom teachers should select and use new technologies in their teaching. Indeed, young people’s lives, particularly during adolescence, are characterised by vast biological, psychological and social changes that influence how they learn and deploy risk and resilient behaviours (Blakemore, 2019). In particular, adolescents’ peer groups are influential. Compared to adults, adolescents are more negatively affected by being socially excluded, more likely to take risks with peers than alone, and more likely to be influenced by the opinions of peers (Blakemore, 2019). These social changes offer an explanation as to why the young people and adults in the sample of this study suggested that peer-based constructivist pedagogies are likely to be effective in supporting and enriching young people’s informal learning through social media.

Taking this analysis further, the ability to take another person’s perspective, the ability to consider the future, and an awareness of self are complex cognitive capacities that are developing during adolescence (Blakemore & Mills, 2014). The potential for cognitive inhibitions in these areas offer another potential explanation as to why the young people in this study positioned teachers as an important resource in helping them to understand the impact of their own and others behaviours on social media. As such, evidence from this study illustrates that teachers will be most effective when they are able to understand and engage with social media as a complex interaction between biological, psychological and social developmental changes.

Looking across the data, it can be argued that collaborative teaching practices and co-construction will help teachers to develop their digital media knowledge and skills. Young people’s uses of social media are highly dynamic and complex, and to respond in ways that are empathetic and pedagogically informed, teachers need to collaborate with their learners in order to understand the subtlety of the rules of engagement. Co-constructing new knowledge in the local context in real time with students is particularly important because time lags in policy development and rapid technological change mean that much of the external advice that reaches teachers is already outdated (Livingstone & Third, 2017). At the same time the evidence from this study points toward the need for more support from education authorities (e.g. governments). Moreover, professional development offered by a range of trusts, organizations and researchers must do more to ensure the latest evidence reaches teachers rapidly. There is a clear need to guarantee that practitioners/professionals have access to the most up-to-date evidence from a range of sub/disciplines (Armour, Quennerstedt, Chambers, & Makopoulou, 2017), and that a variety of methods, tools and resources are deployed to support authentic learning (Avalos, 2011; Carpenter et al., 2016; Cordingly et al., 2015). In schools, and building on well-established characteristics of effective professional development, it can be suggested that schools must focus on supporting teacher agency and capacity building, whereby teachers are prompted, encouraged and supported to critically evaluate evidence, inquire into their practices and develop new insights that are aligned with the needs of their students and their own contexts (Armour, Quennerstedt, Chambers, & Makopoulou, 2017; Avalos, 2011; Cordingly et al., 2015).

Data from this study suggest that schools will be challenged in their attempts to support and build upon young people’s engagement with social media, and to design appropriate and timely teacher professional development. Furthermore, and reinforcing the findings of previous studies (c.f. Carpenter et al., 2016; Carpenter & Krukta, 2014; Galvin & Greenhow, 2020) it was notable in this study that social media was not a priority for most schools, except perhaps to ban it. Yet, based on the perspectives and experiences of young people and the challenges they face on social media platforms in their daily lives, it could be argued that young people have a *right* to be supported by their teachers. Used adeptly, it is also apparent that social media can bring many educational benefits, so schools do need to actively explore this educational potential collaboratively with young people, and in ways that are relative to their on-going social media experiences.

## 7 Conclusion

This paper has reported data from an in-depth qualitative study that examined health-related social media policies and practices in UK schools, and considered the perspectives of young people, teachers and key stakeholders in education and health (researchers, professionals and practitioners). The added value of this research is that new empirical evidence is provided on how and why social media can be positioned as a positive health-related educational resource for young people, and the step changes that are required in schools in order to support teachers to use social media in pedagogically informed ways. It is clear that schools and teachers *do* have an important role in the dynamic digital spaces inhabited by young people but that to engage productively, teachers need to work from an informed position on how and why young people use and navigate social media. Teachers also need to be empathetic rather than antagonistic, and position themselves as ongoing and continuous learners who collaborate with young people to co-construct digital pedagogies.

The evidence reported in this paper supports the need for further empirical, conceptual and theoretical research into the dynamism of young people’s learning across formal (school) and informal (social media) settings, and its rapid evolution over time. Empirically, we need further evidence on the types of pedagogies and teacher professional development that can support teachers to engage with and harness young people’s informal learning through social media. Conceptually, we require analytical frames that can bridge gaps between what technology enables and how it is used, and in ways that acknowledge the reciprocal relationship between learning in formal and informal contexts. Practically, we need new robust and evidence-based pedagogies on the optimal uses of social media in schools and classrooms.

## Funding

This work was supported by Wellcome Trust [grant number 201601/Z/16/Z].

## Footnotes

1 The Office for Standards in Education, Children’s Services and Skills (OfSTED). Is responsible for inspecting services providing education and skills for learners of all ages. Further information can be accessed from: https://www.gov.uk/government/organisations/ofsted.
